# Interrelation between facial soft tissue lessions, underlying fracture patterns and treatment of zygomatic bone trauma: a 10 year retrospective study

**DOI:** 10.1186/s13005-020-00246-y

**Published:** 2020-11-26

**Authors:** Raluca Iulia Juncar, Paul Andrei Tent, Mihai Juncar, Ioan Anton Arghir, Oana Cristina Arghir, Mircea Rivis

**Affiliations:** 1 Department of Oral and Maxillofacial Surgery, University of Oradea, Str. Piața 1 Decembrie, no.10, 410073 Oradea, Romania; 2 Pulmonology Department, Faculty of Medicine, Ovidius University of Constanta, Consteanta, Romania; 3 Discipline of Oral Surgery, 2nd Department of Dental Medicine, “Victor Babeş” University of Medicine and Pharmacy, 2 Eftimie Murgu Sq., 300041 Timisoara, Romania

**Keywords:** Zygomatic fractures, Trauma, Treatment, Clinical patterns, Soft tissue associated lesions

## Abstract

**Background:**

The pattern of zygomatic bone fractures varies in the literature, their features being frequently masked by the presence of associated soft tissue lesions. In this context the clinical diagnosis and the therapeutic indications can be difficult. The aim of this study was to evaluate the clinical features of zygomatic bone fractures and their interrelation with concomitant overlying soft tissue injuries, as well as to assess the type of treatment methods applied depending on the fracture pattern and the results achieved depending on the incidence rate of postoperative complications. We will use these results in order to improve the diagnosis and the establishment of correct treatment of this pathology.

**Methods:**

A 10-year retrospective evaluation of midface fractures was performed in patients diagnosed and treated in a tertiary Clinic of Oral and Maxillofacial Surgery. Statistical analysis was performed with the MedCalc Statistical Software version 19.2 (MedCalc Software bvba, Ostend, Belgium; 53 https://www.medcalc.org; 2020). Nominal data were expressed as frequency and percentage. The comparisons of the frequencies of a nominal variable among the categories of another nominal variable were made using the chi-square test. Multivariate logistic regressions were used in order to establish the independent association between variables and lacerations/excoriations. After using the Bonferroni correction for multiple comparisons, a value of *p* < 0.025 was considered statistically significant.

**Results:**

The study included 242 patients with zygomatic bone fractures. The majority of the fractures were displaced *n* = 179 (73.9%), closed *n* = 179 (73.9%) and complete *n* = 219 (90.5%). Hematoma was the most frequent associated soft tissue lesion *n* = 102 (42.1%) regardless of the fracture pattern (*p* = 1.000). Complete zygomatic fracture (OR – 2.68; *p* = 0.035) and fractures with displacement (OR – 3.66; *p* = 0.012) were independently associated with the presence of laceration. Fractures with displacement (OR – 7.1; *p* = 0.003) were independently associated with the presence of excoriation. The most frequent type of treatment applied was Gillies reduction (61.9%), followed by ORIF (30.9%). The most frequent postoperative complication was malunion secondary to Gillies treatment (4,6%).

**Conclusions:**

Patients presenting lacerations and excoriations on clinical soft tissue examination will most frequently have an underlying complete, displaced or comminuted zygomatic fracture. In the case of displaced, open or comminuted fractures we achieved the best results secondary to ORIF treatment method, while in the case of non-displaced and closed fractures, the best results achieved were secondary to conservative treatment.

## Background

The zygomatic bone through its prominence in the facial contour is more exposed to trauma, being currently the most fractured bone of the midface worldwide [1, 2]. Due to the anatomical relationships of the zygomatic bone with the orbit, the maxillary sinus, the neurocranium, the coronoid process of the mandible and the masticatory muscles, zygomatic fractures may frequently take the form of a complex clinical picture that can always puzzle a less experienced clinician [2]. The complexity of a case is directly proportional with the degree of bone involvement, the degree of displacement, as well as with the relationship with the external environment of the fractured fragments [3]. Concomitant periorbital and perizygomatic soft tissue lesions can be in some cases characteristic of a certain underlying bone fracture pattern, but at the same time they can mask bone fractures with considerable displacement [4]. Hematomas and post-traumatic edema can temporarily compensate the facial asymmetry resulted by the inferomedial displacement of the zygomatic bone [2–4]. They also can prevent adequate palpation of key suture points of the zygomatic bone in the midface and can temporarily mask enophthalmia or diplopia [2–4]. Correct and immediate diagnostic failure of zygomatic bone fractures can lead to incorrect treatment [5]. This can have to severe aesthetic and morphological consequences, difficult to correct secondarily [5]. In this context, conducting a study to highlight the inter-relationship between the type of associated lesions and the underlying fracture pattern will help the medical staff to establish a correct and rapid clinical diagnosis, in which circumstances the rate of long-term complications of this pathology can be reduced. Regardless of the chosen therapeutic method, the objectives of zygomatic bone fracture treatment are: restoring the orbital contour with functional recovery of the globe, opening of the mouth within physiological limits, reduction of the fractured fragments in anatomical position with the restoration of sagittal, vertical and horizontal diameters of the midface and nonetheless the prevention of late complications [1–6]. The therapeutic methods of zygomatic bone fractures include conservative techniques, closed reduction techniques (Gillies, Keen, Caroll-Girard) and open reduction with internal fixation (ORIF) using one up to four point fixation [6]. Although there are currently many specialized publications related to this aspect worldwide, unfortunately there is no consensus about the ideal method of treatment, the opinions of different authors diverging [6, 7]. However, over the past years, a general increase in the preference for ORIF by many authors has been observed [2–7].

Until present no such study was performed in our geographical area, data on this aspect being completely missing. In this context, the aim of this study was to evaluate the clinical features of zygomatic bone fractures and their interrelation with concomitant overlying soft tissue injuries, as well as to assess the type of treatment methods applied depending on the fracture pattern and the quality of the results obtained depending on the incidence rate of postoperative complications. We will use these results in order to improve the rapid diagnosis and the establishment of correct treatment of this pathology.

## Methods

The study was conducted in patients admitted and treated for zygomatic bone fractures in an University Clinic of Oral and Maxillofacial Surgery in Romania, over a 10-year period. The study was approved by the Institutional Review Board (IRB) of Oradea University (IRB no. 1275/14.02.2018) and was therefore performed in accordance with the ethical standards laid down in the 1964 Declaration of Helsinki and its later amendments. All patients included in the study signed an informed consent at the time of their admission to the clinical service, by which they gave their consent to the use of their anonymized medical data for scientific purposes and publication.

The data were extracted from patients’ medical records and the monitored variables were: the degree of bone involvement (incomplete/complete fracture), the degree of bone fragment displacement (displaced/non-displaced/minimally displaced, comminuted), the relationship of the fracture focus with the external environment (closed/intra-orally open/extra-orally open fracture), the type of associated soft tissue lesions (hematoma/excoriation/laceration), type of preoperatively functional impairment (none/diplopia/sensory disturbances in the territory of innervation of the infraorbital nerve/impairment jaw movement), the type of treatment applied (conservative/Gillies/ORIF), the type of postoperative complications. All patients in this study were investigated preoperatively by computed tomography (CT) in order to exclude any intracranial lessions and to identify the pattern of the zygomatic bone fracture. We mention that the term “minimally displaced” is attributed to fractures with less than 2 mm displacement evidenced by CT examination. Also, in our department, closed reduction of zygomatic bone fractures was strictly performed by Gillies temporal approach. Conservative treatment involved non-performance of surgery and patient monitoring for at least 8 weeks. ORIF treatment in our department involves the following surgical approaches: intraoral for the zygomato-alveolar fracture site, infraciliary or preseptal transconjunctival for the inferior orbital rim and in association with lateral canthotomy for fronto-zygomatic fracture site. We mention than in the case of multiple zygomatic fractures the association between this approaches is carried out.

The evaluation of the postoperative evolution was based on the clinical (evaluation of the visual function, amplitude of jaw movements and facial symmetry assessment) and imagistic examination of the patients (CT-scan).

The study definitive inclusion criteria were: presence of at least one fracture line in the zygomatic bone (zigomatic-alveolar suture, temporo-zygomatic suture, fronto-zygomatic suture and inferior orbital rim), an acute trauma episode in the history of the disease, time since trauma less than 14 days, paraclinical examinations (CT examination) confirming the clinical diagnosis of fracture and evidencing its location and characteristics, signing of an informed consent for the use of patient’s medical data for scientific purposes, treatment of the fracture in the study host institution, follow-up of the case for at least 8 weeks postoperatively. We mention that all patients included in this study were older than 10 years of age. Children under the age of 10 are treated in the pediatric surgery service in our region.

Exclusion criteria: patient without fracture lines in the zygomatic bone or its sutures, zygomatic complex fractures associated with orbital fractures, pathological bone fracture, absence of complementary imaging investigations, patient’s refusal to sign an informed consent for the use of his/her medical data for scientific purposes, time since trauma more than 14 days, treatment performed in another service, incomplete data, presence of factors favoring the occurrence of fractures such as bisphosphonate treatment, osteopathies, etc., impossibility of following up the case for at least 8 weeks postoperatively.

Data were centralized in electronic format using Microsoft Excel software. Descriptive statistics of the evaluated cases was performed with a 2 decimal percentage accuracy. Statistical analysis was performed with the MedCalc Statistical Software version 19.2 (MedCalc Software bvba, Ostend, Belgium;53 https://www.medcalc.org; 2020). Nominal data were expressed as frequency and percentage. The comparisons of the frequencies of a nominal variable among the categories of another nominal variable were made using the chi-square test. Multivariate logistic regressions were used in order to establish the independent association between variables and lacerations/excoriations. After using the Bonferroni correction for multiple comaprisons, a value of *p* < 0.025 was considered statistically significant.

## Results

### Degree of bone involvement

The study inclusion criteria were met by 242 patients with zygomatic bone fractures. Regarding bone involvement, the following results were obtained: *n* = 219 (90.50%) had complete zygomatic fractures in all 4 points (zigomatic-alveolar suture, temporo-zygomatic suture, fronto-zygomatic suture and inferior orbital rim), *n* = 21 (8.68%) had complete zygomatic-alveolar, infraorbital and fronto-zygomatic (tripod) fracture lines and an incomplete fracture line in the temporo-zygomatic arch, *n* = 2 (0.83%) had strictly incomplete temporo-zygomatic arch fractures.

### Degree of fracture displacement

Most of the fractures were displaced, *n* = 179 (73.96%), followed by non-displaced/minimally displaced fractures, *n* = 45 (18.61%). Comminuted fractures were in a small proportion, *n* = 18 (7.43%).

### Relashionship with the external environment of the fracture focus

According to the relationship with the external environment, the following results were obtained: *n* = 210 (86.78%) had closed fractures, *n* = 30 (12.40%) had an intra-orally open fracture focus, *n* = 1 (0.41%) had an extra-orally open fronto-zygomatic fracture focus, and *n* = 1 (0.41%) had 2 open fracture foci: an extra-orally open fronto-zygomatic focus and an intra-orally open focus.

### Presence of preoperative functional disorders

The types of preoperative functional disorders were divided in patients as follows: *n* = 98 (40,50%) presented diplopia, limited jaw movement and sensory disturbances all at the same time, *n* = 79 (32,64%) presented both limited jaw movement and sensory disturbances, *n* = 28 (11,57%) presented sensory disturbances only, *n* = 20 (8,26%) presented diplopia only and *n* = 17 (7,02%) present no functional impairment.

### Associated soft tissue injuries

The most frequent associated soft tissue lesion was hematoma, being present in *n* = 198 (82.23%) of all patients. Excoriations were present in *n* = 103 (42.15%) and lacerations in *n* = 76 (31.40%), each percentage being in relation to the total number of affected patients. The incidence of the type of associated soft tissue lesion was correlated with the degree of bone involvement and the degree of fracture fragment displacement (Table 1).

**Table 1:** Incidence of the type of associated soft tissue lesion depending on the degree of bone involvement and fracture displacement

Variables		Hematoma		Laceration		Excoriation	
		No	Yes	No	Yes	No	Yes
Degree of bone involvement	Complete tripod and incomplete TZA fracture	4	19	10	12	8	15
		9.1%	9.6%	6.1%	15.8%	5.8%	14.6%
	Complete zygomatic fracture – 4 points	40	179	155	64	131	88
		90.9%	90.4%	93.9%	84.2%	94.2%	85.4%
P		1.000		0.028		0.037	
Degree of bone displacement	With displacement	38	141	128	50	108	71
		86.4%	71.2%	77.6%	65.8%	77.7%	68.9%
	Comminution	1	17	7	11	3	15
		2.3%	8.6%	4.2%	14.5%	2.2%	14.6%
	Without/Minimal Displacement	5	40	30	15	28	17
		11.4%	20.2%	18.2%	19.7%	20.1%	16.5%
P		0.102		0.015		0.001	

We used a multivariate logistic regression in order to find out which variable is independently associated with the presence of laceration (Table 2). Complete zygomatic fracture (OR – 2.68; *p* = 0.035) and fractures with displacement (OR – 3.66; *p* = 0.012) were independently associated with the presence of laceration.

**Table 2:** Multivariate regression for the presence of lacerations (OR – Odds Ratio; B-regression coefficient)

Variables	B	P	OR	95% C.I. for OR	
				Min	Max
Complete zygomatic fracture – 4 points	0.986	0.035	2.680	1.074	6.692
Without/Minimal Displacement		0.039			
With displacement	1.300	0.012	3.669	1.323	10.172
Comminution	0.301	0.404	1.352	0.666	2.743
Constant	−0.509	0.015	0.601		

We used a multivariate logistic regression in order to find out which variable is independently associated with the presence of excoriation (Table 3). Fractures with displacement (OR – 7.1; *p* = 0.003) were independently associated with the presence of excoriation.

**Table 3:** Multivariate regression for the presence of lacerations (OR – Odds Ratio; B-regression coefficient)

Variables	B	P	OR	95% C.I. for OR	
				Min	Upper
Complete zygomatic fracture – 4 points	0.906	0.057	2.473	0.974	6.279
Without/Minimal Displacement		0.010			
With displacement	1.961	0.003	7.108	1.968	25.675
Comminution	−0.033	0.925	0.968	0.491	1.907
Constant	0.587	0.058	1.799		

### Type of treatment applied

The most frequent treatment method was Gillies temporal approach reduction *n* = 150 (61,98%), followed by ORIF *n* = 75 (30,99%) and conservative treatment *n* = 17 (7,02%). A total number of 225 titanium miniplates 1.7 mm thick and 900 monocortical screws were used. The incidence of the type of treatment was correlated with the relationship of the focus with the external environment and the degree of displacement of the fracture fragments (Table 4).

**Table 4:** Distribution of the type of treatment used depending on the relationship with the external environment and the degree of fracture displacement

		Type of treatment			Total
		Gillies	Conservative	ORIF	
Relationship with the external environment	Closed	150	17	43	210
		100.0%	100%	57,33%	86.78%
	Intra-orally open	0	0	30	30
		0.0%	0.0%	40,00%	12.40%
	Intra-orally and extraorally open	0	0	1	1
		0.0%	0.0%	1,33%	0.41%
	Extra-orally open	0	0	1	1
		0.0%	0,0%	1,33%	0.41%
Total		150	17	75	242
		100.0%	100.0%	100.0%	100.0%
Degree of bone displacement	With displacement	122	0	57	179
		81.3%	0.0%	76.0%	74.0%
	Comminution	0	0	18	18
		0,0%	0.0%	24.0%	7.4%
	Without/Minimal displacement	28	17	0	45
		18.7%	100%	0.0%	18.6%
Total		150	17	75	242
		100.0%	100.0%	100.0%	100.0%

Most of the displaced and closed fractures were reduced by the Gillies method, displaced, comminuted and open fractures were reduced by ORIF, while non-displaced fractures were treated conservatively. This result was statistically significant.

### Evolution and complications

The majority of the cases had a favorable evolution, without reported complications, *n* = 231 (95.50%). The most frequent postoperative complication was malunion, *n* = 7 (2.90%), followed by osteitis in the fracture focus, *n* = 4 (1.60%). The incidence of the type of postoperative complication was correlated with each type of treatment applied (Table 5).

**Table 5:** Distribution of postoperative complications depending on the treatment method used

		Type of treatment			Total
		Gillies	Conservative	ORIF	
Complications	No complications	143	17	71	231
		95,34%	100.0%	85.1%	95.0%
	Osteitis	0	0	4	4
		0.0%	0.0%	5.4%	1.6%
	Malunion	7	0	0	7
		4,66%	0.0%	0,0%	2.9%
		150	17	75	242
		100.0%	100.0%	100.0%	100.0%

Malunion was more frequently found postoperatively, after reduction of the zygomatic bone fracture by the Gillies method, while osteitis in the fracture focus was more frequent after surgical ORIF treatment. These results were statistically significant (*p* = 0.002). We mention that in all 4 patients, osteitis was present strictly in the zygomatic-alveolar fracture focus surgically approached intra-orally. No patient with malunion had significant functional disorders. All patients presented sensory disturbances only classified as infraorbital nerve paresthesia. In this context all 7 patients refused surgical reintervention at their own responsibility. In the case of patients with osteitis in the fracture foci, the osteosynthesis material was removed, zygomatic bone repositioning being unnecessary. Subsequent evolution was favorable in all 4 cases.

## Discussions

The zygomatic bone is one of the pillars of the facial skeleton, having the role and the ability to absorb a large parts of the impact forces developed by a wounding agent [7]. However, when the kinetic energy developed is too high, the zygomatic bone will fracture either as a monoblock, becoming entirely detached from the midface, or in a comminuted manner [7, 8] Depending on the mechanism, the type, the form, the consistency, the surface and the direction of the wounding agent multiple fracture patterns can occur at this level [7, 8]. In our study, complete fractures with zygomatic bone disjunction were predominant, a result supported by other authors as well [2, 4–8]. Incomplete fractures are non-characteristic and rare in the zygomatic complex due to the reduced bone thickness secondary to maxillary sinus pneumatization [9]. In our study, all incomplete fracture lines were found in the temporo-zygomatic arch, either in association with complete anterior tripod fractures of the zygomatic bone or isolated. This result is confirmed in the literature by a number of authors [9–11]. It can be explained by the presence of the cortical bone which is better represented at this level, and also by the fact that the temporo-zygomatic arch can fracture through an indirect flexion mechanism, secondary to direct fracturing and primary inferomedial displacement of the zygomatic bone [10, 11]. In this context, secondary to a low-kinetic energy trauma, the temporo-zygomatic arch fracture is either not synchronous with the anterior suture lines or incomplete [8]. Isolated temporo-zygomatic arch fractures are rare, their reported percentage in the literature ranging between 0.5 and 14% [3–5, 9–11]. A solitary temporo-zygomaic arch fracture, requires a lateral and perpendicular impact direction to the face [10]. Given that the direction of action of wounding agents on the face is most frequently anterior or anterolateral, there are small chances that fractures will occur solely at this level [9–11].

The majority of the fractures in our study were displaced fractures, which is in accordance with the results of other authors [3–5, 9–11]. Contrary to our results, some authors report an increased incidence of non-displaced zygomatic bone fractures [12], while others report a higher incidence of comminuted fractures [13, 14]. Differences between the inclusion criteria in these studies may create the present divergences in the literature. We included exclusively patients with CT imaging investigations based on which we could easily identify minimal displacements of the fractured fragments. Minimal bone fragment displacements may go unnoticed on plain radiographs, the type of fracture being thus classified in a different category [12]. Biomechanically, displacement in the case of zygomatic bone fractures is primary, being directly proportional to the kinetic energy resulting from the impact, the type of the wounding agent and its direction of action [9–11]. Secondary displacement, following traction of the masseter muscle insertions, occurs rarely, having a reduced biomechanically importance [3]. Comminuted fractures occur in the context of strong traumas such as those from road traffic accidents, firearms or explosives [13, 14]. In our study, the small number of comminuted fractures can be attributed to the fact that in our geographical area, injuries caused by interpersonal violence through fist blows are predominant [15]. It is well known that most of the times, the kinetic energy developed by fist blows is rarely sufficient to induce multiple fractures of the viscerocranium [16]. The predominance of injuries to the zygomatic bone through low kinetic energy in our geographical area also explains the high incidence of closed fractures, without communication with the external environment in this study. This is emphasized by the results of other authors [12–17]. The majority of intra-orally open fractures are due to the adherence of the mucoperiosteum to the zygomatic-alveolar ridge, mucoperiosteal laceration occurring secondarily to significant displacement at this level [3–5, 9–11]. In contrast, Keller et al. [13] and Kittle et al. [14], in studies conducted in armed conflict zones, report a predominance of extra-orally open zygomatic bone fractures. The differences in the characteristics of zygomatic bone fractures depending on the studied population, can explain the divergences present in the literature.

Hematoma was the most frequent associated soft tissue lesion in the current study, which is also reported by other authors [2, 4, 18, 19]. Contrary to our results, other authors indicate the highest incidence of laceration [20, 21]. The fact that in the current study hematomas are predominant shows that the severity of the injuries included in this study is reduced.

Gillies temporal approach reduction of the zygomatic bone was the most frequent therapeutic method used in our study, similarly to the results of other authors [2, 22, 23]. The great number of non-comminuted zygomatic fractures in this study explains this result. Although this method is losing ground to the new modern osteosynthesis techniques (ORIF), it can be useful and provide stable results over time in some cases [2]. The advantages of this method are rapid surgery, minimal scar masked by hair, minimal invasiveness, thus avoiding direct opening of the fracture foci, especially of the zygomatic-alveolar focus into the septic oral environment, and implicitly, the decrease in the risk of postoperative osteitis [2]. Another advantage that should not be overlooked is the decrease of hospital costs, the Gillies reduction involving no special materials or equipment, while the duration of hospitalization is minimum [2]. Despite all these advantages, the indications of this method are strictly limited to fractures with reduced displacement, with the integrity of the zygomatic fragment and maintenance of the periosteum on a sufficiently large surface at this level to ensure primary stability of the fractured bone [2]. This is also confirmed by the results of the current study. Some authors completely avoid using this method, saying that maintaining the perfect reduction of the bone fragments is impossible secondary to Gillies approach, which may have consequences over time [3, 6, 7, 17, 24]. Rana et al. [17] indicate the occurance of the zygomatic bone redisplacement at 6 weeks postoperatively following traction of the masseter muscle insertions during functional acts, in all patients in whom ORIF using 3 point fixation was not performed. Contrary to our results, other authors perform zygomatic bone fracture reduction by ORIF, considering it the treatment method of choice for this pathology [3, 6, 7, 17, 24–29]. ORIF of zygomatic bone fractures can be carried out using 1, 2, 3 or very rarely 4 point fixation [3]. We mention that in our study, ORIF was performed using 3 point fixation in all operated patients, according to the current protocol of the study host clinic. In contrast, globally, using the minimum number of fixation points is currently attempted to ensure zygomatic bone immobilization in order to reduce the risk of potential postoperative complications [3, 4, 7, 9].^.^ Forounzanfar et al. [7] and Kim et al. [28] present good results by using ORIF with 1 point fixation, the 3 point fixation of the zygomatic bone being rarely necessary. Czerwinski et al. [5, 29] also report good results after using the endoscopically assisted ORIF-single incision technique. Correct reduction is systematically checked intraoperatively by fluoroscopy or computed tomography [5, 6, 29]. There is currently no consensus regarding the ideal number of fixation points in the case of zygomatic bone fractures, this aspect depending on the fracture pattern, surgeon’s experience and the available means [7].

In zygomatic bone fractures without displacement and without functional disorders, conservative treatment and follow-up of the patient are indicated, approach unanimously accepted in the literature [3, 6, 7, 17, 24–29]. Patient follow-up is absolutely necessary in this context [3, 6, 7, 17, 24–29]. Subsequent zygomatic bone displacement, either secondary to functional movements or due to novo trauma can occur, surgery being required under these conditions [3, 6, 7, 17, 24–29]. In our study, all cases in which conservative treatment was chosen had a favorable evolution. This shows that the indications of this method were entirely respected.

The most frequent postoperative complication was malunion, which occurred postoperatively in the case of Gillies reduction, in accordance with the results of other authors [7]. This result is not surprising given the inaccuracy of this method [9, 17, 18, 25–27]. In contrast, other authors indicate an increased incidence of postoperative osteitis [3, 27]. Osteitis in this study occurred only in 4 cases in the intra-oral osteosynthesis focus, probably due to bacterial contamination from the septic environment of the oral cavity. Removal of the osteosynthesis material under these circumstances led to a favorable result [9, 17, 18, 25–27]. The persistence of infraorbital nerve sensory disturances postoperatively was not considered a postoperative complication in this study, it is known that post-traumatic sensory recovery can last up to 2 years or it may never occur. Also, the sensory disorders were not due to the surgical interventions, but being present clinically preoperatively. This fact is sustained also by other specialized studies [25–30].

The aim of this study was attained. The zygomatic bone fracture pattern and the interrelation between bone lesions and associated soft tissue lesions, as well as the most effective treatment were determined in a significant group of patients. Knowing the frequency of the association between a certain type of soft tissue lesion and a certain underlying zygomatic bone fracture pattern significantly contributes to a rapid and complete diagnosis, the maxillofacial surgeon knowing when to suspect the presence of a fracture masked by other clinical signs.

However, this study has a number of limitations. One of the most important limitations of the current study results from its retrospective nature; the data being collected from patients’ medical records, they might have been incomplete or incorrect. To minimize this drawback, only complete medical records were selected, but in this way, a number of cases from the statistical database were lost. Also the retrospective nature of study cannot allow the same inference as a randomized controlled trial. Therefore a randomized control trial regarding this subject is recommended in the future. Another limitation is the fact that in the midface, zygomatic bone fractures are frequently combined with orbital fractures, being difficult to evaluate retrospectively. Strictly including zygomatic bone fractures in this study resulted in the loss of a significant number of cases.

## Conclusions

Most of the zygomatic bone fractures were complete, closed and with bone fragment displacement. Patients presenting soft tissue lacerations on clinical examination will most frequently have a complete, displaced or comminuted underlying zygomatic fracture. The diagnostic approach of zygomatic bone trauma in our country must be related to this aspect. The best results in the case of displaced, open or comminuted fractures were obtainded secondary to ORIF, while in the case of non-displaced and closed fractures secondary to conservative treatment. The greatest number of postoperative complications occurred secondarily to Gillies temporal reduction, the most frequent being malunion.

## Data Availability

The data and materials are available through e-mail from the corresponding author.
